# The genome sequence of Menetries’ Clouded Yellow, *Colias thisoa* Ménétriès, 1832 (Lepidoptera: Pieridae)

**DOI:** 10.12688/wellcomeopenres.25793.1

**Published:** 2026-01-23

**Authors:** Giorgi Iankoshvili, Jérémy Gauthier, Kay Lucek, Charlotte J. Wright, Joana I. Meier, Mark L. Blaxter

**Affiliations:** 1 Institute of Ecology, Ilia State University, Tbilisi, Tbilisi, Georgia; 2 Naturéum - State Museum of Natural Sciences, Lausanne, Switzerland; 3 University of Neuchâtel, Neuchâtel, Switzerland; 4 Tree of Life Programme, Wellcome Sanger Institute, Hinxton, England, UK

**Keywords:** Colias thisoa; Menetries’ Clouded Yellow; genome sequence; chromosomal; Lepidoptera

## Abstract

We present a genome assembly from a female specimen of *Colias thisoa* (Menetries’ Clouded Yellow; Arthropoda; Insecta; Lepidoptera; Pieridae). The assembly contains two haplotypes with total lengths of 398.28 megabases and 339.66 megabases. Most of haplotype 1 (99.82%) is scaffolded into 33 chromosomal pseudomolecules, including W and Z sex chromosomes and a supernumerary B chromosome. Haplotype 2 was assembled to scaffold level. The mitochondrial genome has also been assembled, with a length of 15.14 kilobases. Gene annotation of this assembly on Ensembl identified 14 651 protein-coding genes. This work is part of Project Psyche, a collaborative programme generating genomes for European butterflies and moths.

## Species taxonomy

Eukaryota; Opisthokonta; Metazoa; Eumetazoa; Bilateria; Protostomia; Ecdysozoa; Panarthropoda; Arthropoda; Mandibulata; Pancrustacea; Hexapoda; Insecta; Dicondylia; Pterygota; Neoptera; Endopterygota; Amphiesmenoptera; Lepidoptera; Glossata; Neolepidoptera; Heteroneura; Ditrysia; Obtectomera; Papilionoidea; Pieridae; Coliadinae; *Colias*; *Colias thisoa* Ménétriès, 1832 (NCBI:txid596544)

## Background

*Colias thisoa* Ménétriés, 1832, known as Menetries’ Clouded Yellow, is a pierid butterfly (Lepidoptera, Pieridae) originating from the high mountains of south-western and central Asia ( Grieshuber, 2014). Its distribution is split into two parts: in the west from Turkey and the Caucasus to northern Iran and in the east from central Asia to the Altai and Sayan ranges of southern Siberia, with a large gap of over 1 000 km between the two ( Grieshuber, 2014). Given its fragmented distribution, several regional subspecies have been described (e.g. *C. thisoa aeolides* Grum-Grshimailo, 1890 from Kyrgyzstan and Tajikistan, *C. thisoa shakuhensis* Sheljuzhko, 1935 from Iran, and *C. thisoa nikolaevi* Korshunov, 1998 from Kazakhstan), but their status is still debated.

Adults are orange-yellow with the characteristic dark marginal band of *Colias* butterflies; males have a narrow unmarked black border, whereas females are a richer orange-red with a broader black band, usually containing small yellow spots. This trait distinguishes *C. thisoa* from related *Colias* (whose females often exhibit pale forms). *Colias thisoa* inhabits subalpine meadows and grassy slopes at elevations of 2 000–3 500 m. The larval host plants include milk-vetches ( *Astragalus* spp.). The species is univoltine with adults flying in summer (mostly June and July). *Colias thisoa* is usually localised, often co-occurring with other montane *Colias* (e.g. *Colias chrysotheme*) on flower-rich upland slopes. *Colias thisoa* has not yet been evaluated by the IUCN. It is considered *Data Deficient* in Turkey’s national Red Book ( Karaçetin & Welch, 2011), but has been protected in Azerbaijan since the 1980s.

*Colias thisoa* belongs to a complex of high-mountain *Colias* with considerable geographic variation and some taxonomic uncertainty. Phylogenetic analyses based on mtDNA barcodes revealed low genetic differentiation among several related species ( Laiho & Ståhls, 2013). This suggests a relatively recent radiation or ongoing gene flow, complicating species delimitation. To date, genetic research on *C. thisoa* has been limited to barcodes. The acquisition of a reference genome for this species will enable in-depth genetic studies to resolve its phylogenetic placement within *Colias* and clarify relationships among subspecies. Moreover, genomic data will also inform conservation status and assess the evolutionary resilience of this butterfly, specialised to live at high altitudes, in the context of global warming.

**Figure 1.**
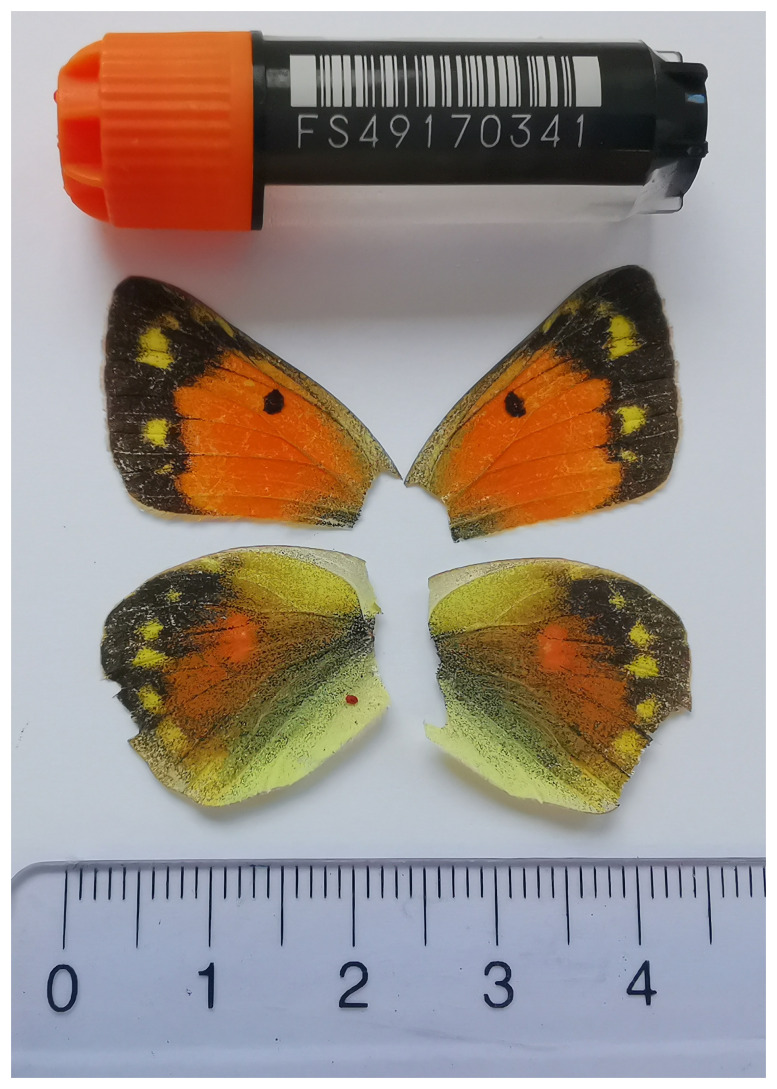
Voucher photograph of the *Colias thisoa* (ilColThis1) specimen used for genome sequencing.

We present a chromosome-level, haplotype-resolved genome sequence for *Colias thisoa*, sequenced as part of Project Psyche. The sequence data were derived from a female specimen ( Figure 1) collected from Stepantsminda, Kazbegi, Georgia.

## Methods

### Sample acquisition

The specimen used for genome sequencing was an adult female *Colias thisoa* (specimen ID SAN28000047, ToLID ilColThis1; Figure 1), collected from Stepantsminda, Kazbegi, Georgia (latitude 42.6617, longitude 44.5928) on 2023-08-26. The specimen was collected and identified by Giorgi Iankoshvili (Ilia State University).

### Nucleic acid extraction

Protocols for high molecular weight (HMW) DNA extraction developed at the Wellcome Sanger Institute (WSI) Tree of Life Core Laboratory are available on protocols.io ( Howard *et al.*, 2025). The ilColThis1 sample was weighed and triaged to determine the appropriate extraction protocol. Tissue from the thorax was homogenised by powermashing using a PowerMasher II tissue disruptor.

HMW DNA was extracted in the WSI Scientific Operations core using the Automated MagAttract v2 protocol. DNA was sheared into an average fragment size of 12–20 kb following the Megaruptor®3 for LI PacBio protocol. Sheared DNA was purified by automated SPRI (solid-phase reversible immobilisation). The concentration of the sheared and purified DNA was assessed using a Nanodrop spectrophotometer and Qubit Fluorometer using the Qubit dsDNA High Sensitivity Assay kit. Fragment size distribution was evaluated by running the sample on the FemtoPulse system. For this sample, the final post-shearing DNA had a Qubit concentration of 41.48 ng/μL and a yield of 1 949.56 ng, with a fragment size of 14.0 kb.

### PacBio HiFi library preparation and sequencing

Library preparation and sequencing were performed at the WSI Scientific Operations core. Libraries were prepared using the SMRTbell Prep Kit 3.0 (Pacific Biosciences, California, USA), according to the manufacturer’s instructions. The kit includes reagents for end repair/A-tailing, adapter ligation, post-ligation SMRTbell bead clean-up, and nuclease treatment. Size selection and clean-up were performed using diluted AMPure PB beads (Pacific Biosciences). DNA concentration was quantified using a Qubit Fluorometer v4.0 (ThermoFisher Scientific) and the Qubit 1X dsDNA HS assay kit. Final library fragment size was assessed with the Agilent Femto Pulse Automated Pulsed Field CE Instrument (Agilent Technologies) using the gDNA 55 kb BAC analysis kit. The sample was sequenced on a Revio instrument (Pacific Biosciences). The prepared library was normalised to 2 nM, and 15 μL was used for making complexes. Primers were annealed and polymerases bound to generate circularised complexes, following the manufacturer’s instructions. Complexes were purified using 1.2X SMRTbell beads, then diluted to the Revio loading concentration (200–300 pM) and spiked with a Revio sequencing internal control. The sample was sequenced on a Revio 25M SMRT cell. The SMRT Link software (Pacific Biosciences), a web-based workflow manager, was used to configure and monitor the run and to carry out primary and secondary data analysis.

Specimen details, sequencing platforms, and data yields are summarised in Table 1.

**Table 1.** Specimen and sequencing data for BioProject PRJEB79435.

Platform	PacBio HiFi	Hi-C
**ToLID**	ilColThis1	ilColThis1
**Specimen ID**	SAN28000047	SAN28000047
**BioSample (source individual)**	SAMEA115109508	SAMEA115109508
**BioSample (tissue)**	SAMEA115434398	SAMEA115434397
**Tissue**	thorax	head
**Instrument**	Revio	Illumina NovaSeq X
**Run accessions**	ERR13615500	ERR13621431
**Read count total**	1.00 million	662.78 million
**Base count total**	8.84 Gb	100.08 Gb

### Hi-C

***Sample preparation and crosslinking***

The Hi-C sample was prepared from 20–50 mg of frozen tissue from the head of the ilColThis1 sample using the Arima-HiC v2 kit (Arima Genomics). Following the manufacturer’s instructions, tissue was fixed and DNA crosslinked using TC buffer to a final formaldehyde concentration of 2%. The tissue was homogenised using the Diagnocine Power Masher-II. Crosslinked DNA was digested with a restriction enzyme master mix, biotinylated, and ligated. Clean-up was performed with SPRISelect beads before library preparation. DNA concentration was measured with the Qubit Fluorometer (Thermo Fisher Scientific) and Qubit HS Assay Kit. The biotinylation percentage was estimated using the Arima-HiC v2 QC beads.

***Hi-C library preparation and sequencing***

Biotinylated DNA constructs were fragmented using a Covaris E220 sonicator and size selected to 400–600 bp using SPRISelect beads. DNA was enriched with Arima-HiC v2 kit Enrichment beads. End repair, A-tailing, and adapter ligation were carried out with the NEBNext Ultra II DNA Library Prep Kit (New England Biolabs), following a modified protocol where library preparation occurs while DNA remains bound to the Enrichment beads. Library amplification was performed using KAPA HiFi HotStart mix and a custom Unique Dual Index (UDI) barcode set (Integrated DNA Technologies). Depending on sample concentration and biotinylation percentage determined at the crosslinking stage, libraries were amplified with 10–16 PCR cycles. Post-PCR clean-up was performed with SPRISelect beads. Libraries were quantified using the AccuClear Ultra High Sensitivity dsDNA Standards Assay Kit (Biotium) and a FLUOstar Omega plate reader (BMG Labtech).

Prior to sequencing, libraries were normalised to 10 ng/μL. Normalised libraries were quantified again to create equimolar and/or weighted 2.8 nM pools. Pool concentrations were checked using the Agilent 4200 TapeStation (Agilent) with High Sensitivity D500 reagents before sequencing. Sequencing was performed using paired-end 150 bp reads on the Illumina NovaSeq X.

Specimen details, sequencing platforms, and data yields are summarised in Table 1.

### Genome assembly

Prior to assembly of the PacBio HiFi reads, a database of *k*-mer counts ( *k* = 31) was generated from the filtered reads using FastK. GenomeScope2 ( Ranallo-Benavidez *et al.*, 2020) was used to analyse the *k*-mer frequency distributions, providing estimates of genome size, heterozygosity, and repeat content.

The HiFi reads were assembled using Hifiasm in Hi-C phasing mode ( Cheng *et al.*, 2021; Cheng *et al.*, 2022), producing two haplotypes. Hi-C reads ( Rao *et al.*, 2014) were mapped to the primary contigs using bwa-mem2 ( Vasimuddin *et al.*, 2019). Contigs were further scaffolded with Hi-C data in YaHS ( Zhou *et al.*, 2023), using the --break option for handling potential misassemblies. The scaffolded assemblies were evaluated using Gfastats ( Formenti *et al.*, 2022), BUSCO ( Manni *et al.*, 2021) and MERQURY.FK ( Rhie *et al.*, 2020).

The mitochondrial genome was assembled using MitoHiFi ( Uliano-Silva *et al.*, 2023), which runs MitoFinder ( Allio *et al.*, 2020) and uses these annotations to select the final mitochondrial contig and to ensure the general quality of the sequence.

### Assembly curation

The assembly was decontaminated using the Assembly Screen for Cobionts and Contaminants ( ASCC) pipeline. TreeVal was used to generate the flat files and maps for use in curation. Manual curation was conducted primarily in PretextView and HiGlass ( Kerpedjiev *et al.*, 2018). Scaffolds were visually inspected and corrected as described by Howe *et al*. (2021). Manual corrections included 15 breaks and 249 joins. This reduced the scaffold count by 66.7%, increased scaffold N50 by 4.3%, and increased the total assembly length by 5.0%. The curation process is described at https://gitlab.com/wtsi-grit/rapid-curation. PretextSnapshot was used to generate a Hi-C contact map of the final assembly.

### Assembly quality assessment

The Merqury.FK tool ( Rhie *et al.*, 2020), run in a Singularity container ( Kurtzer *et al.*, 2017), was used to evaluate *k*-mer completeness and assembly quality for both haplotypes using the *k*-mer database ( *k* = 31) computed prior to genome assembly. The analysis outputs included assembly QV scores and completeness statistics.

The genome was analysed using the BlobToolKit pipeline, a Nextflow ( Di Tommaso *et al.*, 2017) implementation of the earlier Snakemake version ( Challis *et al.*, 2020). The pipeline aligns PacBio reads using minimap2 ( Li, 2018) and SAMtools ( Danecek *et al.*, 2021) to generate coverage tracks. It runs BUSCO ( Manni *et al.*, 2021) using lineages identified from the NCBI Taxonomy ( Schoch *et al.*, 2020). For the three domain-level lineages, BUSCO genes are aligned to the UniProt Reference Proteomes database ( Bateman *et al.*, 2023) using DIAMOND blastp ( Buchfink *et al.*, 2021). The genome is divided into chunks based on the density of BUSCO genes from the closest taxonomic lineage, and each chunk is aligned to the UniProt Reference Proteomes database with DIAMOND blastx. Sequences without hits are chunked using seqtk and aligned to the NT database with blastn ( Altschul *et al.*, 1990). The BlobToolKit suite consolidates all outputs into a blobdir for visualisation. The BlobToolKit pipeline was developed using nf-core tooling ( Ewels *et al.*, 2020) and MultiQC ( Ewels *et al.*, 2016), with containerisation through Docker ( Merkel, 2014) and Singularity ( Kurtzer *et al.*, 2017).

We painted Merian elements, which represent the 32 ancestral linkage groups in Lepidoptera ( Wright *et al.*, 2024), along chromosomes using lep_busco_painter. Painting used BUSCO gene locations from the lepidoptera_odb10 set ( Kriventseva *et al.*, 2019) and chromosome lengths from NCBI Datasets. Each complete BUSCO (including both single-copy and duplicated BUSCOs) was assigned to a Merian element using a reference database. Positions were coloured and plotted along chromosomes drawn to scale.

## Genome sequence report

### Sequence data

**Figure 2.**
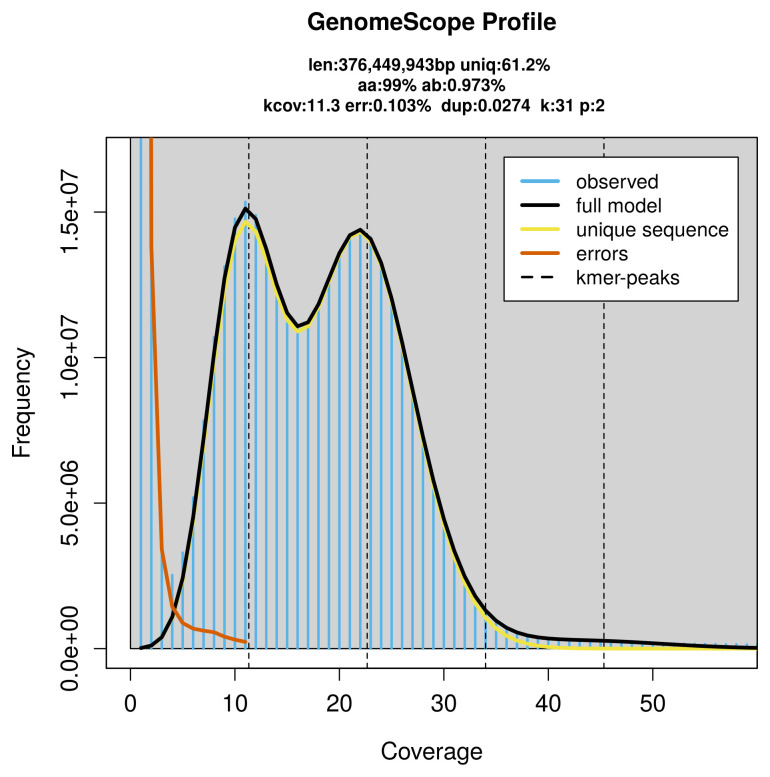
Frequency distribution of *k*-mers generated using GenomeScope2. The plot shows observed and modelled *k*-mer spectra, providing estimates of genome size, heterozygosity, and repeat content based on unassembled sequencing reads.

PacBio sequencing of the *Colias thisoa* specimen generated 8.84 Gb (gigabases) from 1.00 million reads, which were used to assemble the genome. GenomeScope2.0 analysis estimated the haploid genome size at 377.11 Mb, with a heterozygosity of 0.97% and repeat content of 38.79% ( Figure 2). These estimates guided expectations for the assembly. Based on the estimated genome size, the sequencing data provided approximately 23× coverage. Hi-C sequencing produced 100.08 Gb from 662.78 million reads, which were used to scaffold the assembly. Table 1 summarises the specimen and sequencing details.

### Assembly statistics

The genome was assembled into two haplotypes using Hi-C phasing. Haplotype 1 was curated to chromosome level, while haplotype 2 was assembled to scaffold level. The final assembly has a total length of 398.28 Mb in 59 scaffolds, with 343 gaps, and a scaffold N50 of 12.47 Mb ( Table 2).

**Table 2.** Genome assembly statistics.

**Assembly name**	ilColThis1.hap1.1	ilColThis1.hap2.1
**Assembly accession**	GCA_964275215.1	GCA_964275285.1
**Assembly level**	chromosome	scaffold
**Span (Mb)**	398.28	339.66
**Number of chromosomes**	33	scaffold-level
**Number of contigs**	402	226
**Contig N50**	3.24 Mb	3.28 Mb
**Number of scaffolds**	59	71
**Scaffold N50**	12.47 Mb	12.3 Mb
**Longest scaffold length (Mb)**	23.12	-
**Sex chromosomes**	W and Z	-
**Organelles**	Mitochondrion: 15.14 kb	-

**Figure 3.**
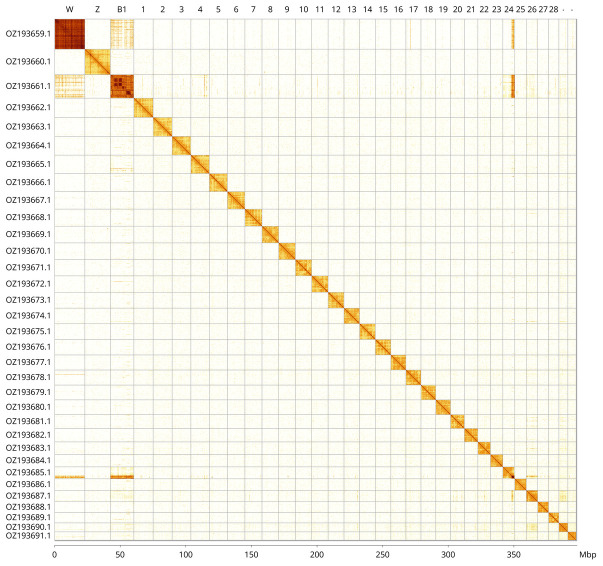
Hi-C contact map of the *Colias thisoa* genome assembly. Assembled chromosomes are shown in order of size and labelled along the axes, with a megabase scale shown below. The plot was generated using PretextSnapshot.

**Figure 4.**
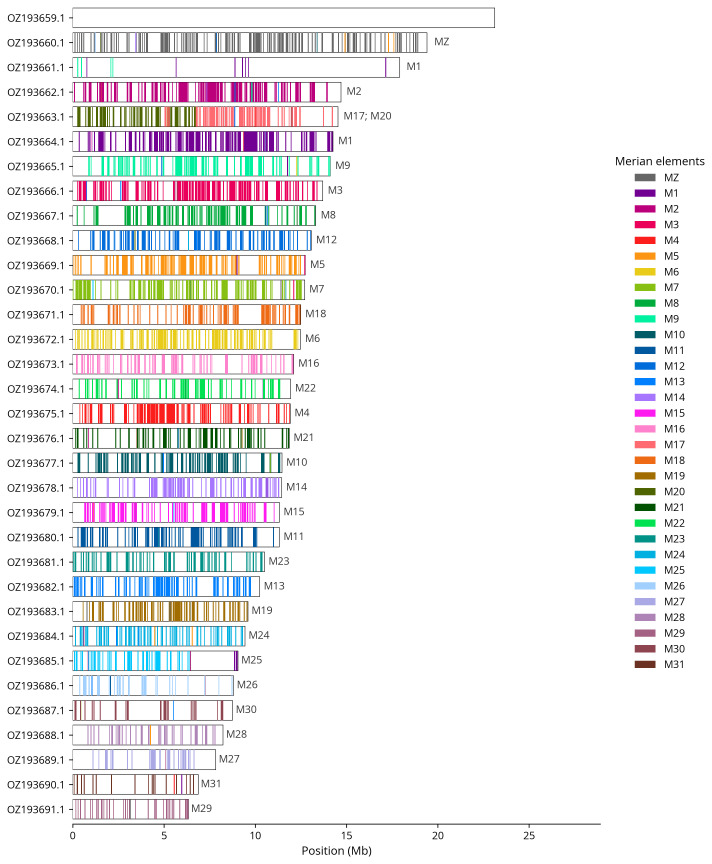
Merian elements painted across chromosomes in the ilColThis1.hap1.1 assembly of *Colias thisoa*. Chromosomes are drawn to scale, with the positions of orthologues shown as coloured bars. Each orthologue is coloured by the Merian element that it belongs to. All orthologues which could be assigned to Merian elements are shown.

Most of the assembly sequence (99.82%) was assigned to 33 chromosomal-level scaffolds, representing 30 autosomes and the B _1_, W, and Z chromosomes. These chromosome-level scaffolds, confirmed by Hi-C data, are named according to size ( Figure 3; Table 3). Chromosome painting with Merian elements illustrates the distribution of orthologues along chromosomes and highlights patterns of chromosomal evolution relative to Lepidopteran ancestral linkage groups ( Figure 4). Chromosome Z and W were assigned based on Hi-C signal. During curation, we noted that the order and orientation of scaffolds making up chromosomes B _1_ and W are uncertain.

**Table 3.** Chromosomal pseudomolecules in the haplotype 1 genome assembly of *Colias thisoa* ilColThis1.

INSDC accession	Molecule	Length (Mb)	GC%	Assigned Merian elements
OZ193662.1	1	14.70	34	M2
OZ193663.1	2	14.54	34	M17;M20
OZ193664.1	3	14.26	34	M1
OZ193665.1	4	14.10	33.50	M9
OZ193666.1	5	13.70	34	M3
OZ193667.1	6	13.31	33.50	M8
OZ193668.1	7	13.06	33.50	M12
OZ193669.1	8	12.74	33.50	M5
OZ193670.1	9	12.70	33.50	M7
OZ193671.1	10	12.49	33.50	M18
OZ193672.1	11	12.47	33.50	M6
OZ193673.1	12	12.11	33.50	M16
OZ193674.1	13	11.93	33.50	M22
OZ193675.1	14	11.91	33.50	M4
OZ193676.1	15	11.86	33.50	M21
OZ193677.1	16	11.46	33.50	M10
OZ193678.1	17	11.45	34	M14
OZ193679.1	18	11.32	33.50	M15
OZ193680.1	19	11.31	34	M11
OZ193681.1	20	10.51	34	M23
OZ193682.1	21	10.25	33.50	M13
OZ193683.1	22	9.60	34	M19
OZ193684.1	23	9.44	33.50	M24
OZ193685.1	24	9.05	37	M25
OZ193686.1	25	8.80	33.50	M26
OZ193687.1	26	8.75	35.50	M30
OZ193688.1	27	8.23	33.50	M28
OZ193689.1	28	7.83	33.50	M27
OZ193690.1	29	6.88	35	M31
OZ193691.1	30	6.35	34	M29
OZ193659.1	W	23.12	33	-
OZ193660.1	Z	19.41	34	MZ
OZ193661.1	B1	17.92	35.50	M1

The mitochondrial genome was also assembled (length 15.14 kb, OZ193692.1). This sequence is included as a contig in the multifasta file of the genome submission and as a standalone record.

### Assembly quality metrics

**Figure 5.**
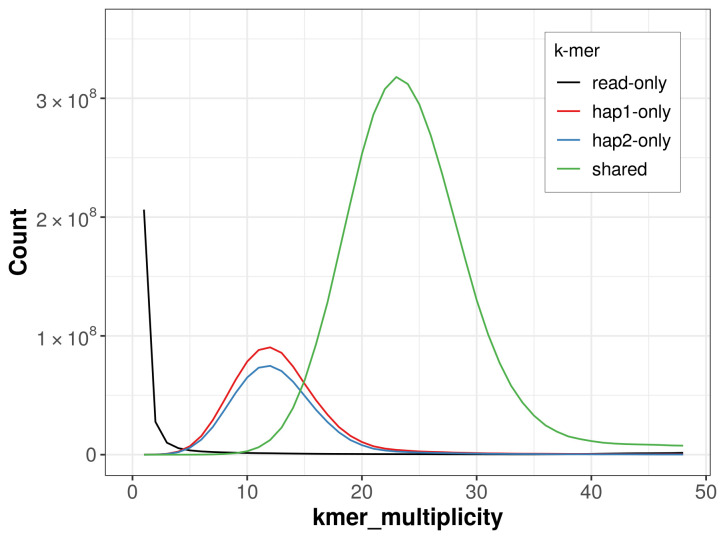
Evaluation of *k*-mer completeness using MerquryFK. This plot illustrates the recovery of *k*-mers from the original read data in the final assemblies. The horizontal axis represents *k*-mer multiplicity, and the vertical axis shows the number of *k*-mers. The black curve represents *k*-mers that appear in the reads but are not assembled. The green curve (the homozygous peak) corresponds to *k*-mers shared by both haplotypes and the red and blue curves (the heterozygous peaks) show *k*-mers found only in one of the haplotypes.

**Figure 6.**
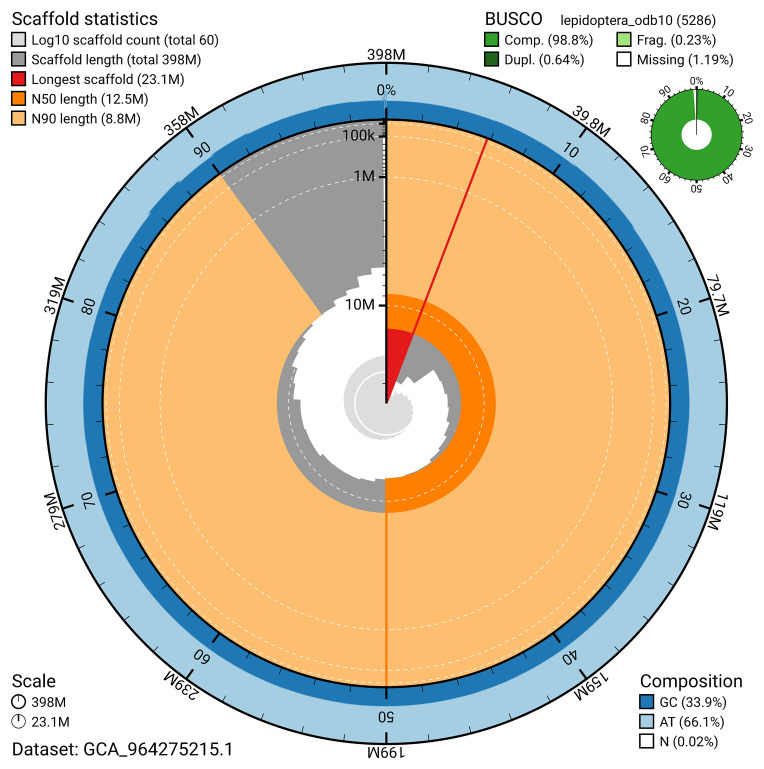
Assembly metrics for ilColThis1.hap1.1. The BlobToolKit snail plot provides an overview of assembly metrics and BUSCO gene completeness. The circumference represents the length of the whole genome sequence, and the main plot is divided into 1,000 bins around the circumference. The outermost blue tracks display the distribution of GC, AT, and N percentages across the bins. Scaffolds are arranged clockwise from longest to shortest and are depicted in dark grey. The longest scaffold is indicated by the red arc, and the deeper orange and pale orange arcs represent the N50 and N90 lengths. A light grey spiral at the centre shows the cumulative scaffold count on a logarithmic scale. A summary of complete, fragmented, duplicated, and missing BUSCO genes in the set is presented at the top right. An interactive version of this figure can be accessed on the BlobToolKit viewer.

**Figure 7.**
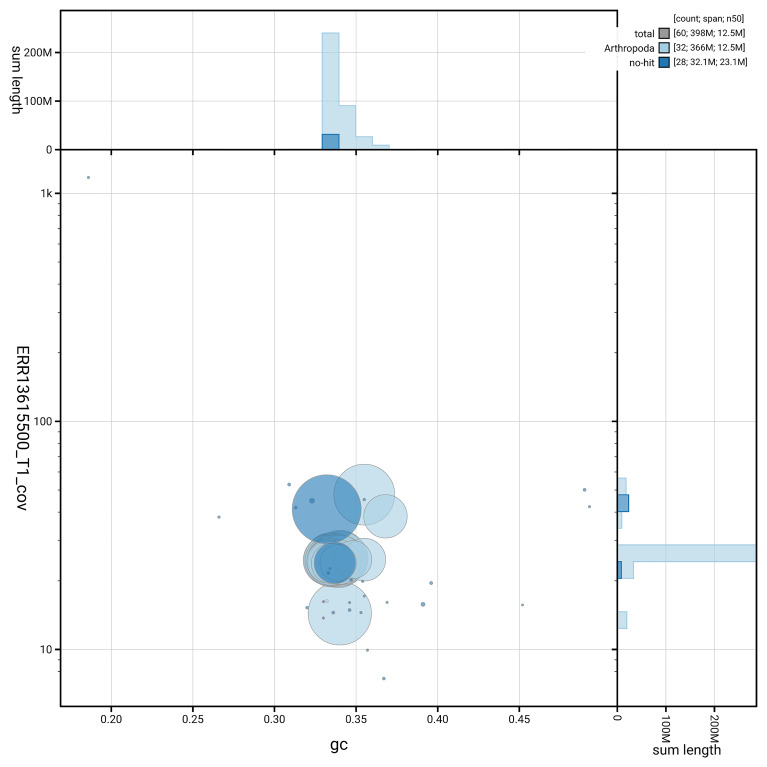
BlobToolKit GC-coverage plot for ilColThis1.hap1.1. Blob plot showing sequence coverage (vertical axis) and GC content (horizontal axis). The circles represent scaffolds, with the size proportional to scaffold length and the colour representing phylum membership. The histograms along the axes display the total length of sequences distributed across different levels of coverage and GC content. An interactive version of this figure is available on the BlobToolKit viewer.

For haplotype 1, the estimated QV is 60.8, and for haplotype 2, 63.2. When the two haplotypes are combined, the assembly achieves an estimated QV of 61.7. The *k*-mer completeness is 80.30% for haplotype 1, 76.19% for haplotype 2, and 98.44% for the combined haplotypes ( Figure 5). BUSCO analysis using the lepidoptera_odb10 reference set ( *n* = 5 286) identified 98.8% of the expected gene set (single = 98.2%, duplicated = 0.6%) in haplotype 1. For haplotype 2, BUSCO analysis identified 94.6% of the expected gene set (single = 94.3%, duplicated = 0.3%). The snail plot in Figure 6 summarises the scaffold length distribution and other assembly statistics for haplotype 1. The blob plot in Figure 7 shows the distribution of scaffolds by GC proportion and coverage for haplotype 1.

Table 4 lists the assembly metric benchmarks adapted from Rhie *et al.* (2021) the Earth BioGenome Project Report on Assembly Standards September 2024. The EBP metric, calculated for the haplotype 1, is **6.C.Q60**, meeting the recommended reference standard.

**Table 4.** Earth Biogenome Project summary metrics for the *Colias thisoa* assembly.

Measure	Value	Benchmark
EBP summary (haplotype 1)	6.C.Q60	6.C.Q40
Contig N50 length	3.24 Mb	≥ 1 Mb
Scaffold N50 length	12.47 Mb	= chromosome N50
Consensus quality (QV)	Haplotype 1: 60.8; haplotype 2: 63.2; combined: 61.7	≥ 40
*k*-mer completeness	Haplotype 1: 80.30%; Haplotype 2: 76.19%; combined: 98.44%	≥ 95%
BUSCO	C:98.8% [S:98.2%; D:0.6%]; F:0.2%; M:1.0%; n:5 286	S > 90%; D < 5%
Percentage of assembly assigned to chromosomes	99.82%	≥ 90%

**Notes:** EBP summary uses log10(Contig N50); chromosome-level (C) or log10(Scaffold N50); Q (Merqury QV). BUSCO: C=complete; S=single-copy; D=duplicated; F=fragmented; M=missing; n=orthologues

### Genome annotation report

The *Colias thisoa* genome assembly (GCA_964275215.1) was annotated by Ensembl at the European Bioinformatics Institute (EBI). This annotation includes 37 779 transcribed mRNAs from 14 651 protein-coding and 8 257 non-coding genes. The average transcript length is 13 817.25 bp, with an average of 1.65 coding transcripts per gene and 6.62 exons per transcript. For further information, please refer to the Ensembl annotation page.

### Wellcome Sanger Institute – Legal and Governance

The materials that have contributed to this genome note have been supplied by a Tree of Life collaborator. The Wellcome Sanger Institute employs a process whereby due diligence is carried out proportionate to the nature of the materials themselves, and the circumstances under which they have been/are to be collected and provided for use. The purpose of this is to address and mitigate any potential legal and/or ethical implications of receipt and use of the materials as part of the research project, and to ensure that in doing so, we align with best practice wherever possible. The overarching areas of consideration are:

- Ethical review of provenance and sourcing of the material
- Legality of collection, transfer and use (national and international).

Each transfer of samples is undertaken according to a Research Collaboration Agreement or Material Transfer Agreement entered into by the Tree of Life collaborator, Genome Research Limited (operating as the Wellcome Sanger Institute), and in some circumstances, other Tree of Life collaborators.

## Data Availability

European Nucleotide Archive: Colias thisoa. Accession number PRJEB79435. The genome sequence is released openly for reuse. The *Colias thisoa* genome sequencing initiative is part of the Sanger Institute Tree of Life Programme (PRJEB43745) and Project Psyche (PRJEB71705). All raw sequence data and the assembly have been deposited in INSDC databases. Raw data and assembly accession identifiers are reported in Table 1 and Table 2. Pipelines used for genome assembly at the WSI Tree of Life are available at https://pipelines.tol.sanger.ac.uk/pipelines. Table 5 lists software versions used in this study.

## Author information

Contributors are listed at the following links:

- Members of the Wellcome Sanger Institute Tree of Life Management, Samples and Laboratory team
- Members of Wellcome Sanger Institute Scientific Operations – Sequencing Operations
- Members of the Wellcome Sanger Institute Tree of Life Core Informatics team
- Members of the Tree of Life Core Informatics collective
- Members of the Project Psyche Community
