# Supplementary material for: The genome sequence of Menetries’ Clouded Yellow, Colias thisoa Ménétriès, 1832 (Lepidoptera: Pieridae)
Source: Wellcome Open Res. 2026 Jan 23;11:64. [Version 1] doi: 10.12688/wellcomeopenres.25793.1 (PMC12914173; doi:10.12688/wellcomeopenres.25793.1)
Supplement: Peer review history

# Reviewer report: Reviewer response for version 1

**DOI:** 10.21956/wellcomeopenres.28394.r146634

**Published:** 2026-03-16

**Authors:** Fedor Čiampor

**Affiliations:** 1 Department of Biodiversity and Ecology, Plant Science and Biodiversity Centre, Slovak Academy of Sciences, Bratislava, Slovakia

The manuscript "The genome sequence of Menetries’ Clouded Yellow, Colias thisoa Ménétriès, 1832 (Lepidoptera: Pieridae)“ presents a high-quality chromosome-level genome assembly for the butterfly Colias thisoa, produced within the Tree of Life / Project Psyche initiative. The work is technically sound, clearly documented, and valuable as a genomic resource for evolutionary, taxonomic, and conservation research on high-mountain Lepidoptera. The paper fits well as a data note. The methodology and results are described concisely and clearly. The  results indicate a robust reference genome suitable for future comparative genomics and population studies.

 The manuscript is largely technical, I would acknowledge at least brief exploratory analyses why this species, what other related species were already sequenced etc. Also the geographic description could be clearer in relation to the European-focused project, and the ecological context could better motivate the importance of the species. 

 Despite few comments, this is a well-executed genome resource paper providing a high-quality reference assembly with excellent technical standards and full reproducibility.

Are sufficient details of methods and materials provided to allow replication by others?

Yes

Is the rationale for creating the dataset(s) clearly described?

Yes

Are the datasets clearly presented in a useable and accessible format?

Yes

Are the protocols appropriate and is the work technically sound?

Yes

Reviewer Expertise:

(e)DNA metabarcoding, invertebrate diversity

I confirm that I have read this submission and believe that I have an appropriate level of expertise to confirm that it is of an acceptable scientific standard.

# Reviewer report: Reviewer response for version 1

**DOI:** 10.21956/wellcomeopenres.28394.r145749

**Published:** 2026-02-17

**Authors:** Ram Hari Chapagain

**Affiliations:** 1 Kanti Children’s Hospital, Kathmandu, Nepal

Genome assembly from a female Colias thisoa (Lepidoptera) produced two haplotypes of 398.28 Mb and 339.66 Mb. Haplotype 1 (99.82%) scaffolded into 33 chromosomal pseudomolecules including W, Z and a B chromosome; haplotype 2 scaffold-level. Mitochondrial genome 15.14 kb. Ensembl annotation identified 14,651 protein-coding genes with good methodology and proprer discussion .

Are sufficient details of methods and materials provided to allow replication by others?

Yes

Is the rationale for creating the dataset(s) clearly described?

Yes

Are the datasets clearly presented in a useable and accessible format?

Yes

Are the protocols appropriate and is the work technically sound?

Yes

Reviewer Expertise:

paediatrican

I confirm that I have read this submission and believe that I have an appropriate level of expertise to confirm that it is of an acceptable scientific standard.

# Reviewer report: Reviewer response for version 1

**DOI:** 10.21956/wellcomeopenres.28394.r146637

**Published:** 2026-02-13

**Authors:** Matthew Merkin

**Affiliations:** 1 University of Liverpool, Liverpool, UK

The authors present a chromosome-level genome assembly for the Menetries’ Clouded Yellow butterfly ( *Colias thisoa*) as part of Project Psyche. DNA was extracted from the thorax and head to obtain HiFi and HiC sequencing reads respectively. The genome was then assembled, scaffolded and manually corrected to produce an assembly with 30 autosomes, Z, W and B chromosomes. QC of this assembly showed a BUSCO completeness of 98.8% and an EBP score of 6.C.Q60, suggesting that it is highly suitable for use as a reference genome. The genome was also annotated with 14651 protein coding genes. 

 Comments:

 I find it misleading to report that there were 33 chromosomal pseudomolecules in the abstract. This is fine in the assembly statistics section, but it gives off the impression that n=33 i.e. 32 autosomes and a sex chromosome. It would perhaps be better to explicitly state that this 33 number includes 30 autosomes as well as the Z,W and B.

 Perhaps it is worth rephrasing the first sentence about the range of this butterfly being entirely in Asia, especially since Project Psyche aims to sequence European lepidopterans. Although Turkey and Georgia are mentioned later on in the background, it could help to start by saying they are found in the Caucasus region instead of south-western Asia. 

 There is little discussion of the B chromosome in this genome. For instance, the introduction could mention its importance for investigating the dynamics of supernumerary chromosomes. The results could also briefly describe the difference in coverage between the B and the autosomes and the annotation report could briefly describe the number of genes on the B chromosome.

Are sufficient details of methods and materials provided to allow replication by others?

Yes

Is the rationale for creating the dataset(s) clearly described?

Yes

Are the datasets clearly presented in a useable and accessible format?

Yes

Are the protocols appropriate and is the work technically sound?

Yes

Reviewer Expertise:

comparative genomics, entomology, genome assembly, museomics

I confirm that I have read this submission and believe that I have an appropriate level of expertise to confirm that it is of an acceptable scientific standard.
